# Factors influencing the use of reproductive health services among young women in Nepal: analysis of the 2016 Nepal demographic and health survey

**DOI:** 10.1186/s12978-020-00954-3

**Published:** 2020-06-29

**Authors:** Naba Raj Thapa

**Affiliations:** Department of Population Studies, Ratna Rajyalaxmi Campus, Tribhuvan University, Kathmandu, Nepal

**Keywords:** Antenatal care, Contraceptive use, Reproductive health services, Skilled birth attendants, Young women

## Abstract

**Background:**

Utilization of reproductive health services is a key component for preventing young women from different sexual and reproductive health problems. Thus, the objective of this study is to determine the factors influencing the use of reproductive health services among young women in Nepal.

**Methods:**

Data have been extracted from the 2016 Nepal Demographic and Health Survey (NDHS) datasets wherein the weighted sample population size was restricted for modern contraceptive use to 1593 whereas for the antenatal care and skilled birth attendants to1606. This study has selected three reproductive health indicators as outcome variables of reproductive health service utilization for the analysis viz, modern contraceptive use, at least four antenatal care visits, and use of skilled birth attendants. Likewise, all calculations are based on standard sample weight of NDHS.

**Results:**

The study has found that 21% of young women used modern contraception, 71% attended at least four ANC visits, and 67% utilized a skilled birth attendant at delivery. Young Janajati women, women having 1–2, and 3 or more living children, women participating household decision-making, and the ones having exposure to media were more likely to use modern contraceptives, whereas the women who want more children were less likely to use them. Higher education attainments, higher wealth quintile, and lower birth order were associated with higher level of receiving at least four ANC visits and SBAs. However, the young women willing to have more children and having access to media have higher odds of receiving at least four ANC visits; and the women attending four and more ANC visits have higher odds of using SBAs.

**Conclusions:**

In order to improve the use of reproductive health services among young women, efforts should be made to enrich the young women of lower educational level, lower economic status, higher birth order, and lower exposure to media. Further research is required to detect the causes that affect the use of reproductive health services.

## Plain English summary

Reproductive health is one of the most important components of human health, and poor reproductive health has more severe consequences in women. The studies and resources on factors associated with the use of reproductive health services among young women in Nepal are limited. Therefore, this study assesses the factors influencing the use of reproductive health services by young women in Nepal using the data from the 2016 Nepal Demographic and Health Survey. The study includes population of married women aged 15–24 years. The sample population for contraceptive use is 1593 and for antenatal care (ANC) visits and skilled birth attendant (SBA) is 1606.

About 21% of young women used modern contraceptive methods. Likewise, 71% of the young women attended at least four ANC visits and 67% used SBA.. Furthermore, the number of living children, fertility preference, women’s participation in household decision-making and exposure to media were some important factors influencing modern contraceptive use. Besides, women’s education, working status, wealth quintile, birth order, fertility preference and exposure to media were the main determiners of at least four ANC visits. Similarly, women’s education, wealth quintile, birth order and ANC visits were the main factors influencing the use of SBAs.

From this perspective, efforts should be made, targeting the young women of lower educational level and economic status, lower exposure to media and higher birth order to increase the utilization of the reproductive health services in Nepal.

## Background

In Nepal, about one-fifth of the total population comprises young people aged 15–24 years [1]. These young people are one of the important sub-groups of population from the concern of reproductive health. It is obvious that young people are facing diverse sexual and reproductive health challenges like unintended pregnancy, pregnancy related health care and sexually transmitted infections including HIV [2, 3]. Moreover, young women aged 15–24 years are at high risk of complications of pregnancy and death [4].

The reproductive health is the basic aspect of overall health [5] in both women and men. A broader concept of reproductive health focuses on contraceptive choices, maternal and newborn health cares, sexually transmitted infections and sexual health [6]. The increasing use of reproductive health services helps reduce maternal mortality and improve the health of children and women. To reduce global maternal mortality and ensure universal access to sexual and reproductive healthcare services are the targets of Sustainable Development Goal (SDG) 3 [7]. For achieving the targets of SDG 3, improving women’s sexual and reproductive healthcare services are essential.

The International Conference on Population and Development (ICPD) 1994 defines that reproductive healthcare is the constellation of methods, techniques and services that contribute to reproductive health and well-being by preventing and solving reproductive health problems [8]. The reproductive healthcare service includes family planning counselling, services for prenatal care, safe delivery and postnatal care, prevention and appropriate care related to reproduction and sexually transmitted diseases [5].

Before the ICPD held in Cairo in 1994, adolescent and youth specific reproductive health services were not existent in Nepal [9]. With the growing concern on reproductive health, the Government of Nepal developed the National Reproductive Health Strategy (1998) for providing integrated reproductive health services. Besides this, the National Adolescent Health and Development Strategy (2000) was also introduced to enhance the health and socioeconomic status of adolescent and youth.

The use of contraception can prevent unwanted pregnancy, early childbearing, and abortion related death. Although the knowledge of family planning is almost universal among adolescent and youth, the use of modern contraceptive is low among young women [10, 11]. Antenatal Care (ANC) is a key component to monitor and reduce the risk of mortality among young mothers during pregnancy and childbirth. Likewise, skilled birth attendance (SBA) is another important factor in reducing maternal and neonatal mortality. Nevertheless, only 21% of married young women aged 15–24 were found using modern contraceptives and about 72% were found receiving four or more ANC visits [12].

The government of Nepal has termed young women as a vulnerable and underserved group to accomplish the specific service required to meet their particular problems and needs. Independent studies conducted in Ethiopia [13, 14], Bangladesh [15, 16] and Nepal [17] have revealed that reproductive health service utilization among adolescent and youth was low and the use of reproductive health services was associated with different socio-demographic and economic factors. In Nepal, there are limited studies regarding the use of reproductive health services among young women. In this connection, this study was carried out to determine the factors influencing the use of reproductive health services among young women in Nepal.

## Data and methods

### Data sources

This study has utilized the data from the 2016 Nepal Demographic and Health Survey (NDHS) datasets. The NDHS is a nationally representative cross-sectional survey conducted under the aegis of the Ministry of Health of the Government of Nepal. In the 2016 NDHS, 11473 households were selected as samples of which 11,040 were interviewed. Similarly, from among 13,089 women aged 15–49 identified for individual interviews, only 12,862 women were successfully interviewed with the response rate of 98%. To select the household for the survey, stratified two-stage cluster sampling was used in rural areas whereas three-stage in urban ones. In rural areas, wards were selected as primary sampling units (PSUs) in the first stage and the households were finalized in the second stage. In urban areas, wards were selected as PSUs in the first stage, one enumeration area (EA) was selected from each PSU in the second stage, and households were selected from sample EAs in the third stage. Next, face-to-face interviews of eligible women aged 15–49 in the sample households were conducted with structured questionnaires.

There were six questionnaires administered in the 2016 NDHS: the household questionnaire, the women’s questionnaire, the men’s questionnaire, the biomarker questionnaire, the fieldworker questionnaire and the verbal autopsy questionnaire. This study used the data collected through women’s questionnaire which was utilized to collect information from among the married women aged 15–49 years. The women’s questionnaire sought the information of background characteristics, reproductive history and child mortality, knowledge and use of family planning methods, fertility preferences, delivery care, child health, women’s works, antenatal delivery and postnatal care, husband’s background characteristics and domestic violence [18]. The Individual Record (IR) data file was employed for this study. We confined the analyses to the 1593 young women who were currently married and non-pregnant for modern contraceptive use and 1606 young women who had birth in the 5 years preceding the survey for ANC visits and SBA for their most recent birth.

### Variables

#### Dependent variables

Here, three reproductive health indicators were selected as dependent variables for the analysis. The first dependent variable is the current use of modern contraception which is dichotomous. Young women who were currently using modern contraception were coded as 1 and otherwise as 0. The modern contraceptive methods include male and female sterilization, injectables, intrauterine devices, pills, implants, male condoms, locational amenorrhea, and emergency contraceptives.

The second dependent variable is ANC visits for the most recent pregnancy in the 5 years preceding the survey, which is also dichotomous. The number of ANC visits were coded as 1 if women attended four or more ANC visits before their most recent birth and coded as 0 if there were fewer than four visits. Then the third dependent variable is the assistance of the SBA which is also dichotomous. Here, women assisted by the SBA during most recent delivery were coded as 1 and otherwise as 0. The SBAs include doctors, nurses, and midwives [18, 19].

#### Independent variables

The independent variables of this study are women’s age, education, ethnicity, occupation, wealth quintile, birth order, number of living children, participation in household decision-making and exposure to media. The age groups of women in this study are 15–19 and 20–24. Mother’s age at birth was classified into two age groups- < 20 and 20–24. Education level achieved by young women was classified into four categories: no education, primary, some secondary, and SLC and above. Moreover, caste/ethnicity variable was categorized into five groups- Brahman/Chhetri, Terai caste, Dalit, Janajati, and Muslim/other. Women’s working status was grouped into two categories- not working, and working. In the same way, wealth quintile variable was recorded into five categories- poorest, poor, middle, richer, and richest. Wealth quintile is a composite measure of household economic status composed of key household assets. Household wealth quintile was constructed using data of household assets such as televisions, bicycles, materials used for house construction, sources of drinking water, sanitation facilities and other wealth related characteristics [20]. Birth order was measured in three categories- 1, 2, and 3 or more. Similarly, the number of living children was measured in three categories- 0, 1–2, and 3 or more whereas fertility preference of young women was categorized into three groups- wants no more children, wants more children, and undecided. Women’s participation in household decision-making was studied in terms of three decisions: women’s own healthcare, large household purchases, and visit to family or relatives. Each of these three aspects of decision-making had five response options: respondent alone, respondent and husband/partner jointly, husband/partner, someone else and other. These responses were coded as 1 for yes (respondent alone, and respondent and husband/partner jointly) and 0 for no (husband/partner, someone else, and other). Then, a single composite variable was constructed by grouping women into two categories: women who participated in at least one decision were categorized as participated one or more decisions (=1) otherwise as not participated (=0). Media exposure is dichotomous variable. Finally, women exposed to either radio, television or newspapers/magazines at least once a week were coded as exposed (=1) and those exposed to none as not exposed (=0).

#### Methods of data analysis

This study employed three levels of data analyses. Firstly, univariate analysis was carried out to analyze the selected socio-demographic characteristic of young women aged 15–24. Secondly, the chi-square test was carried out to examine the association between the modern contraceptive use, at least four ANC visits and the use of SBA, and explanatory variables such as socio-demographic, economic, women’s participation in household decision-making and media exposure. Third, multivariate analysis with logistic regression was performed to examine the factors influencing the use of reproductive health services. Likewise, factors significantly associated with outcome variables at *p* < 0.05 were included in multivariate logistic regression. Multicollinearity assessment was employed before the multivariate analysis. The results were shown in adjusted odds ratios (OR) with 95% confidence interval (CI). The data were analyzed by using STATA version 15.1. All calculations were weighted using standard sample weight of NDHS. The STATA survey command ‘svy’ was applied to adjust for stratified sample design.

## Results

### Background characteristics of young women

Table 1 shows that more than half of young women were aged 15–19. Forty percent of young women completed the secondary level of education. About 43% of young women were not working but 57% were working. About 21% of young women lived in Province 2 and 20% lived in Bagmati Province. Janajati (36%) and Brahman/Chhetri (29%) were dominant castes/ethnicities. Nearly 18% of young women were from the poorest, 21% from the middle, and 18% from the richest wealth quintile. 32% young women had 1–2 living children and 68% had exposure to media. Overall, the majority of young women were aged 15–19, educated, working, Janajati and Brahman/Chhetri and exposed to media.

**Table 1:** Percent distribution of young women aged 15–24 by background characteristics, Nepal DHS 2016

Background characteristics	Percent	Number
**Age**		
15–19	53.6	2598
20–24	46.4	2251
**Education level**		
No education	10.0	483
Primary	14.3	696
Secondary	40.3	1953
SLC & above	35.4	1718
**Working status**		
Not working	42.7	2070
Working	57.4	2779
**Province**		
Province 1	16.8	817
Province 2	20.5	996
Bagmati	19.6	952
Gandaki	9.5	460
Province 5	18.4	892
Karnali	6.0	293
Sudurpashchim	9.1	440
**Ethnicity**		
Brahman/Chhetri	29.3	1420
Terai caste	15.2	737
Dalit	13.2	641
Janajati	36.2	1755
Muslim	6.1	296
**Wealth quintile**		
Poorest	17.5	847
Poorer	20.5	994
Middle	20.9	1015
Richer	22.8	1104
Richest	18.3	890
**Number of living children**		
0	66.0	3202
1–2	31.8	1543
3+	2.2	104
**Exposure to media (at least once a week)**		
Not exposed	31.9	1548
Exposed	68.1	3301
**Total**	**100.0**	**4849**

### Bivariate analysis

Table 2 presents the relationship between modern contraceptive use and selected background characteristics. Here, less than one quarter of young women were found to have used modern contraception. The results revealed that the independent variables were significantly associated with modern contraceptive use at *p* < 0.05. Modern contraceptive use was positively associated with age group. The use of contraception was higher among women aged 20–24 than among women aged 15–19. In this study, young women’s education and wealth quintile were insignificantly related to modern contraceptive use. Young women, who were better educated, working, Janajati, wealthier and having three or more living children; who wanted no more children, participated household decision making and had exposure to media had the highest level of contraceptive use. Moreover, use of modern contraception was the lowest among young women who didn’t have education and exposure to media; were in the middle wealth quintile, Muslim and not working; and did not participate household decision-making.

**Table 2:** Percentage of young women aged 15–24 using modern contraception according to background characteristics, Nepal DHS 2016

	Modern contraceptive use			
Background characteristics	Percent	95% CI	Number	***p***-value (χ^**2**^)
**Age**				
15–19	14.5	[11.5–18.1]	704	0.000
20–24	23.9	[21.3–26.8]	1684	
**Education**				
No education	15.9	[12.1–20.6]	396	0.085
Primary	20.4	[16.4–25.0]	486	
Secondary	23.0	[19.6–26.7]	914	
SLC & above	22.4	[18.5–26.7]	593	
**Working status**				
Not working	17.8	[14.8–21.2]	1041	0.006
Working	23.7	[21.0–26.7]	1347	
**Caste/ethnicity**				
Brahman/Chhetri	22.4	[18.8–26.5]	608	0.000
Terai caste	13.5	[9.6–18.7]	463	
Dalit	17.4	[12.9–23.0]	366	
Janajati	28.6	[24.7–33.0]	795	
Muslim	9.3	[5.4–15.6]	157	
**Wealth quintile**				
Poorest	22.5	[18.8–26.7]	440	0.422
Poorer	22.8	[18.4–27.9]	526	
Middle	18.7	[15.1–22.9]	589	
Richer	19.6	[15.6–24.4]	548	
Richest	23.9	[18.2–30.8]	286	
**Number of living children**				
0	8.5	[6.3–11.4]	754	0.000
1–2	26.0	[23.2–29.0]	1532	
3+	42.0	[30.4–54.6]	103	
**Fertility preference**				
Wants no more children	33.6	[29.7–37.6]	745	0.000
Wants more children	15.1	[12.9–17.8]	1540	
Undecided	20.9	[13.4–31.0]	103	
**Women’s participation in decision-making**				
Not participated	16.2	[13.9–18.8]	1226	0.000
Participated one or more decisions	26.4	[23.2–29.7]	1162	
**Exposure to media (at least once a week)**				
Not exposed	17.7	[14.8–21.0]	946	0.005
Exposed	23.4	[20.7–26.4]	1443	
**Total**	**21.1**	**[18.9–23.5]**	**2389**	

The associations between ANC visits and skilled birth attendant for most recent delivery according to selected background characteristics have been presented in Table 3. About 72% of young women made at least four ANC visits for the most recent birth. The bivariate analysis demonstrated that the independent variables, excluding age of women, were significantly associated (p < 0.05) with at least four ANC visits. The Mothers aged below 20 years at birth appeared more likely to make at least four ANC visits (73%). ANC visits varied as per women’s education. 54% young women with no education made at least four ANC visits compared with 87% young women who attained SLC and above education. A large proportion of young women who are working (78%) reported at least four ANC visits. Besides, there is significant relationship between caste/ethnicity and ANC visits. A greater proportion of young Brahman/Chhetri women made at least four ANC visits while young Muslim women had a small proportion. Young women in the richest wealth quintile (80%) were more likely to make at least four ANC visits than those in the poorest wealth quintile (65%). The percentage of young women making at least four ANC visits decreased as birth order increased. Level of ANC visits was higher among those who wanted more children (77%), participated in household decision making (75%), and had exposure to media (79%).

**Table 3:** Percentage of young women aged 15–24 who had at least four visits for ANC and SBA for most recent birth in the 5 years preceding the survey according to background characteristics, Nepal DHS 2016

	4+ ANC visits			Skilled birth attendant			
Background characteristics	%	95% CI	***p***-value (χ^**2**^)	%	95% CI	***p***-value (χ^**2**^)	Number
**Mother’s age at birth**							
< 20	73.2	[68.7–77.3]	0.268	68.6	[64.0–72.8]	0.177	792
20–25	70.2	[65.7–74.4]		65.0	[60.8–69.0]		813
**Education**							
No education	54.1	[45.7–62.2]	0.000	52.2	[44.3–60.0]	0.000	307
Primary	63.0	[56.5–69.0]		56.6	[49.8–63.2]		350
Secondary	76.9	[72.4–80.9]		72.5	[67.7–76.9]		599
SLC & above	86.9	[82.9–90.1]		79.9	[74.9–84.1]		349
**Working status**							
Not working	65.3	[59.7–70.6]	0.000	69.3	[64.7–73.5]	0.104	707
Working	76.7	[73.1–80.0]		64.8	[60.5–68.9]		899
**Caste/ethnicity**							
Brahman/Chhetri	82.2	[77.6–86.0]	0.000	72.3	[66.1–77.8]	0.033	399
Terai caste	63.6	[53.5–72.6]		62.9	[55.4–69.8]		334
Dalit	67.2	[59.4–74.1]		57.9	[51.1–64.3]		241
Janajati	73.3	[67.8–78.3]		68.2	[61.7–74.1]		517
Muslim	60.7	[48.6–71.6]		70.9	[59.7–80.0]		114
**Wealth quintile**							
Poorest	64.6	[57.9–70.8]	0.007	45.5	[39.0–52.1]	0.000	314
Poorer	70.1	[64.1–75.5]		58.5	[52.1–64.6]		371
Middle	69.9	[62.9–76.0]		73.6	[67.7–78.7]		397
Richer	77.6	[71.4–82.8]		76.2	[70.7–80.9]		355
Richest	80.2	[71.5–86.8]		88.9	[80.7–93.9]		169
**Birth order**							
1	78.7	[75.3–81.8]	0.000	74.7	[71.1–78.1]	0.000	1034
2	61.3	[55.1–67.2]		55.8	[49.6–61.8]		434
3+	51.6	[42.1–61.0]		41.5	[31.8–51.8]		138
**Fertility preference**							
Wants no more children	64.1	[59.1–68.7]	0.000	62.8	[58.1–67.2]	0.005	685
Wants more children	76.9	[73.0–80.5]		69.1	[65.1–72.9]		854
Undecided	82.7	[70.2–90.6]		77.8	[65.9–86.3]		67
**Women’s participation in decision-making**							
No	68.7	[64.1–73.0]	0.021	64.0	[59.6–68.2]	0.045	783
One or more decisions	74.6	[70.4–78.4]		69.3	[65.0–73.4]		810
**Exposure to media (at least once a week)**							
Not exposed	62.5	[56.7–68.0]	0.000	56.9	[52.2–61.4]	0.000	687
Exposed	78.5	[75.0–81.7]		74.2	[69.9–78.1]		919
**Total**	**71.7**	**[68.1–75.0]**		**66.8**	**[63.3–70.1]**		**1606**

Table 3 reflects the use of SBA for the most recent birth according to selected background variables. About 69% of young women used SBA in delivery. It was found that all background variables except mother’s age at birth and working status had a statistically significant association (*p* < 0.05) with SBA. Mothers aged below 20 years at birth used SBA more often than those aged 20–24 years. The percentage of young women using SBA increased with the increase in women’s education level from 52% among young women with no education to 80% among young women with SLC and higher education. A large proportion of young Brahman/Chhetri women (72%) used SBA. It was found that the use of SBA was positively related to the wealth quintile and negatively related to the birth order. Young women who were desirous of more children, participated in household decision making, and had exposure to media were more likely to use SBA.

### Multivariate analysis

Table 4 presents the factors correlated with the use of modern contraception among currently married young women aged 15–24. In the multivariate analysis, women’s education and wealth quintile were excluded because they were not found to be significant associations with modern contraceptives use in bivariate analysis. The results of multivariate logistic regression demonstrate that young women who were Janajati, having 1–2 and 3 or more living children, participated one or more household decisions, and had exposure to media were more likely to use modern contraception. The young Janajati women were found 1.76 times more likely to use modern contraception than young Dalit women. The odds of modern contraceptives use were 3.22 times and 9.07 times greater for women with 1–2, and 3 or more children respectively compared to young women having no living children. The young women who wanted more children were less likely to use modern contraception (OR = 0.57, *p* < 0.001) compared with young women who wanted no more children. The odds of modern contraceptive use were 1.34 times greater among young women who participated one or more household decisions and 1.49 times greater among young women who had exposure to media compared with young women who did not participate in household decisions and had no exposure to media respectively.

**Table 4:** Odds ratios from logistic regression showing factors associated with the use of modern contraception among young women aged 15–24, Nepal DHS 2016

Background variable	Odds ratio	95% confidence interval
**Age**		
15–19	1.00	
20–24	0.98	0.71–1.34
**Working status**		
Not working	1.00	
Working	1.12	0.85–1.48
**Caste/ethnicity**		
Dalit	1.00	
Brahman/Chhetri	1.30	0.84–2.02
Terai caste	0.64	0.36–1.16
Janajati	1.76**	1.15–2.70
Muslim	0.52	0.25–1.05
**Number of living children**		
0	1.00	
1–2	3.22***	2.31–4.49
3+	9.07***	4.88–16.86
**Fertility preference**		
Wants no more children	1.00	
Wants more children	0.57***	0.45–0.73
Undecided	0.71	0.41–1.24
**Women’s participation in decision-making**		
Not participate	1.00	
Participate one or more decisions	1.34*	1.04–1.74
**Exposure to media (at least once a week)**		
Not exposed	1.00	
Exposed	1.49**	1.15–1.92

**Note:** *** *p* < 0.001, ** *p* < 0.01, * *p* < 0.05

Table 5 illustrates those factors associated with at least four ANC visits and SBA at delivery for most recent birth among young married women aged 15–24. Mother’s age at birth was excluded from multivariate analysis because this variable was insignificantly associated with ANC visits. The results of logistic regression show that education (secondary, and SLC and above), working status, wealth quintile (middle, richer and richest), second birth order, young women who wanted more children, and exposure to media had significant association with attending at least 4 ANC visits. The analysis indicates that the odds of attending at least four ANC visits were 1.93 times greater among young women with secondary education compared with young women with no education; and 2.91 times greater among young women with SLC and higher education. The young working women were 1.90 times more likely to make at least four ANC visits, compared with nonworking ones. The young women in the richer wealth quintile were 2.55 times more likely to attend at least four ANC visits than young women in the poorest wealth quintile whereas young women in the richest and the middle wealth quintile appeared 2.12 and 1.85 times more likely to make at least four ANC visits than the women in the poorest wealth quintile respectively. The odds of receiving ANC visits were 36% lower for women with second-order birth compared to women with first-order birth. The young women wanting more children were 1.65 times more likely to make at least four ANC visits compared to young women expecting no more children. The young women with exposure to media were 48% more likely to receive at least four ANC visits compared to those with no exposure.

**Table 5:** Odds ratios from logistic regression showing factors associated with ANC visit (4 + ANC visits) and SBA among young women aged 15–24, Nepal DHS 2016

	4+ ANC Visit		Skilled birth attendant	
Background variable	Odds ratios	95% CI	Odds ratio	95% CI
**Education**				
No education	1.00		1.00	
Primary	1.25	0.85–1.83	1.14	0.77–1.68
Secondary	1.93***	1.31–2.84	1.59*	1.03–2.44
SLC & above	2.91***	1.79–4.76	1.35	0.84–2.16
**Working status**				
Not working	1.00			
Working	1.90***	1.43–2.53		
**Caste/ethnicity**				
Dalit	1.00		1.00	
Brahman/Chhetri	1.29	0.81–2.07	1.22	0.77–1.93
Terai caste	0.78	0.45–1.36	0.76	0.45–1.28
Janajati	0.83	0.53–1.31	1.04	0.73–1.48
Muslim	0.69	0.37–1.29	1.18	0.59–2.37
**Wealth quintile**				
Poorest	1.00		1.00	
Poorer	1.30	0.88–1.91	1.69**	1.19–2.40
Middle	1.85*	1.16–2.97	4.46***	2.70–7.38
Richer	2.55***	1.54–4.21	4.01***	2.56–6.27
Richest	2.12*	1.14–3.93	9.16***	4.47–18.76
**Birth order**				
1	1.00		1.00	
2	0.64**	0.46–0.88	0.49***	0.35–0.67
3+	0.70	0.45–1.08	0.35***	0.21–0.58
**Fertility preference**				
Wants no more children	1.00		1.00	
Wants more children	1.65***	1.25–2.18	0.86	0.65–1.12
Undecided	1.89	0.92–3.89	1.01	0.50–2.07
**Women’s participation in decision-making**				
Not participate	1.00		1.00	
Participate one or more decisions	1.12	0.87–1.44	1.16	0.89–1.52
**Exposure to media (at least once a week)**				
Not exposed	1.00		1.00	
Exposed	1.48*	1.09–2.02	1.21	0.90–1.63
**ANC visits**				
Less than four visits			1.00	
Four or more visits			2.59***	1.95–3.43

Note: *** *p* < 0.001, ** *p* < 0.01, * *p* < 0.05

Table 5 also depicts the results of the logistic regression of young women’s use of SBA during delivery for most recent birth. Mother’s age at birth and women’s working status were excluded from multivariate logistic analysis due to insignificant association with SBA. The analysis shows that the odds of receiving SBA were 1.59 times greater among young women with secondary education compared to young women with no education and 1.35 times greater among young women with SLC and higher education. The odds of SBA increase as the level of wealth quintile rises. The young women in the richest wealth quintile had 9.16 times higher odds of receiving SBA compared to young women in the poorest wealth quintile. Birth order seemed to be another one of the most important predictors of SBA. Higher order births had lower odds of SBA. The odds of receiving SBA were 65% lower for three and more birth orders compared to first order birth and 51% lower for second order birth. The young women making at least four ANC visits had 2.59 times higher odds of receiving SBA compared to those making less than four ANC visits.

## Discussion

The use of modern contraception (21%) by young women was lower than currently married women in the age of 15–49 years (43%). Among young women, the proportion of those receiving at least four ANC visits (72%) was higher compared to the ones in the 15–49 age group (69%). Similarly, the proportion of young women assisted by SBA for the most recent birth (69%) was higher than the national average (61%).

This study shows that inequalities in contraceptive use among young women occurred in terms of caste/ethnicity. The young Jananati women had higher odds of modern contraceptive use compared to young Dalit women. Studies conducted in Nepal revealed that odds of using modern contraceptive were higher for Janajati women compared to other caste/ethnic groups [12, 21]. It is argued that differences in religious faith, and cultural belief and values between caste/ethnic groups may be some possible reasons for disparities in the use of contraception. There is a significant positive association between the number of living children and the use of modern contraceptives. Young women with more living children were more likely to use modern contraceptives that those with less living children. This finding is in line with the previous studies from Ghana [22], Malawi [23], and Ethiopia [24]. This may be argued that women with more children may reach the desired family size so that they are more likely to use contraceptives than women with no or less living children. Fertility preference has been identified as an important factor to influence the use of modern contraceptives. That is, young women who want more children are less likely to use modern contraceptives than women who want no more children. This finding is consistent with different studies conducted in Ethiopia [25], Bangladesh [26], Nepal [11, 21] and Nigeria [27]. This study has also found that young women who participate in household decision making have higher odds of using modern contraceptives than the ones not participating in such decision making process. This finding is consistent with previous studies that have shown a strong association between women’s participation in household decision making and use of modern contraceptives [24, 27, 28]. Such a situation might take place because educated and employed women are primarily involved in household decision making and may have more liberty to use contraceptives.

Exposure to media was found to have a positive association with modern contraceptive use [22, 29, 30]. Television, radio and newspapers were most commonly used sources of information regarding contraceptives in Nepal [31]. This study also indicates that young women exposed to media are more likely to use modern contraceptives. The reason behind this may be the women with access to mass media keep good knowledge on reproductive health problems and services which helps them in improving their knowledge of and build confidence towards using modern contraceptives.

The results from this study have revealed that higher educational level of mothers, higher wealth quintile, and exposure to media are positively associated with at least four ANC visits while higher birth order is negatively associated. These findings are consistent with studies conducted in Indonesia [32], India [33], and Nepal [11]. The study also found that young women who want more children are more likely to receive ANC than the ones who do not. This finding is consistent with the study undertaken in Benin [34]. Women’s working status is found to be a significant predictor of using ANC as young working women are more likely to attend at least four ANC visits than not working ones. This finding is supported by a previous study conducted in Ethiopia [35].

Our finding suggests that the women’s education level is an important variable affecting the use of SBA because young women with secondary and higher education are more likely to get assisted by SBAs during delivery. This finding is consistent with previous studies conducted in Nepal [36] and Ghana [37]. Likewise, this study has found that the use of SBA is inequitable in Nepal. Women with low wealth quintile are less likely to use SBAs. This is in line with the result of previous studies conducted in the South Asian Region [38] and Bangladesh [39]. Also, the finding on birth order and SBAs is consistent with the result of a study conducted in the South Asian Region [38] and India [40], as young women with high birth order are less likely to seek SBAs. The finding of this study revealed that the use of ANC is strongly associated with using SBA among young women. Young women who attend at least four ANC visits are more likely to have SBAs in delivery. This findings is consistent with previous studies in Nepal [36], and Ghana [37].

The possible explanation of this relationship can indicate that educated women may have acquired more knowledge about reproductive health and better understanding of benefits of ANC and SBAs; as a result, they have a higher level of ANC visits and a higher chance of having SBAs at delivery. Women in the richest wealth quintile are more likely to be more educated and have the financial capacity to afford the ANC visits and to seek the help of the SBAs in delivery. But mothers of first-order birth have no experience of childbirth, they fear from the possible complication of childbirth. Thus, they are more likely to receive ANC and use SBAs. Meanwhile, this practice has declined subsequent births due to the experience of pregnancy and childbirth. Exposure to media may increase women’s knowledge or awareness and positive attitude towards ANC and SBAs. As a result, young women who read newspapers, listen to the radio and watch television are more likely to use reproductive health services. In relation to women’s working status, those who are working are more likely to be educated, involve in household decision making and get access to reproductive health services than those who are not. The ANC visits help young women have better understanding of risk in regard to the complication of pregnancy and childbirth and enable them to make a decision regarding the use of SBAs [41].

### Limitations and strengths

This study is based on the data of a cross-sectional survey from the 2016 NDHS. Therefore, this study is limited to establishing causal relationships between variables. However, efforts were made to examine the association between explanatory variables and outcome variables. This study has included those variables which were available for analysis of DHS. Another limitation is that this study does not include time series data in the analysis. The main strength of the study is the use of nationally representative data and multivariate methods to identify factors influencing the use of reproductive health services among young women.

## Conclusions

To sum up, there has been significant improvement in reproductive health indicators among young women. The finding of this study has revealed that number of living children, fertility preference, women’s participation in household decision-making and exposure to media are the important determiners of modern contraceptive use among young women in Nepal. Therefore, family planning programs should focus on young women of low parity and those who want more children. This study has also identified women’s education, working status, wealth quintile, birth order, fertility preference and exposure to media as factors influencing of ANC visits. Likewise, the study has shown that women’s education, wealth quintile, birth order, and ANC visits significantly influence the use of SBAs in delivery among young women. Efforts to encourage attending at least four ANC visits and taking assistance of SBAs should be targeted at young women having lower educational and economic status. The study has also indicated that improving women’s education level, generating employment opportunities, improving economic status, proper media campaigns and encouraging them to attend at least four ANC visits are important steps to increase the use of reproductive health services in Nepal. In order to expand the use of reproductive health services among young women, necessary efforts should be made targeting young women of lower educational level, economic status and exposure to media, and higher birth order. Finally, the results of the study have indicated that further research is required to understand the perspective of young women towards reproductive health service thereby exploring the barriers of the use of reproductive health services.

## Data Availability

The data used in this study are freely available from The DHS Program website (https://www.dhsprogram.com/data/available-datasets.cfm). The study was carried out specifically using 2016 Nepal Demographic and Health Survey dataset.

## Abbreviations

ANC: Antenatal Care
CI: Confidence Interval
DHS: Demographic and Health Survey
EA: Enumeration Area
NDHS: Nepal Demographic and Health Survey
OR: Odds Ratio
PSU: Primary Sampling Unit
SBA: Skilled Birth Attendant
SDG: Sustainable Development Goal
SLC: School Leaving Certificate
